# A mixed methods study of health care professionals’ attitudes towards vaccination in 15 countries

**DOI:** 10.1016/j.jvacx.2022.100219

**Published:** 2022-09-21

**Authors:** Abrar Alasmari, Heidi J. Larson, Emilie Karafillakis

**Affiliations:** a Department of Infectious Disease Epidemiology, London School of Hygiene and Tropical Medicine, London, United Kingdom; b Department of Health Metrics Sciences, University of Washington, Seattle, United States; c Centre for the Evaluation of Vaccination, Vaccine & Infectious Disease Institute, University of Antwerp, Antwerp, Belgium

**Keywords:** Vaccine hesitancy, Healthcare professionals, COVID-19 vaccine, Influenza vaccine, Europe

## Abstract

**Background:**

Health care professionals are widely considered to be the most trusted source of information on vaccine-related topics. However, several are reporting their own hesitancy around certain vaccines, influencing their intention to vaccinate themselves as well as influencing their recommendations to their patients and target population.

**Methods:**

A mixed-methods approach was used including an online survey (n = 1,504) in 15 countries which aimed to determine drivers of HCPs vaccine confidence and examine how these drivers vary across nations. Thirty in-depth semi-structured interviews were conducted with 10 HCPs in a subset of three countries (France, Greece and Hungry) to explore barriers to HCPs vaccine uptake and their role in addressing vaccine hesitancy among patients.

Findings.

The survey’s regression analysis identified that nurses/midwives and HCPs from Hungary, Italy, Romania and Switzerland were less confident in the safety, importance or effectiveness of vaccines in general. Morocco (35%), Turkey (53%) and Greece (69%) reported the lowest influenza vaccination coverage among HCPs. Morocco also reported the lowest rates of HCPs who were “highly likely” to recommend MMR vaccine (34%), HPV vaccine (31%) and Covid-19 vaccines (29%). More than third of HCPs reported a lack of trust in health authorities and in the information they provide. Thematic analysis revealed that concerns over the risk of side-effects associated with vaccines, preference for natural immunity, whether it was necessary to be vaccinated against influenza every year, not having any chronic disease risk factors, and vaccines mandates as the key barriers to HCPs vaccination against influenza and Covid-19.

**Conclusion:**

HCPs have an important role in vaccination and their confidence in vaccination and health authorities must be improved as this may affect their uptake of vaccines and influence their recommendations to their patients. Investigating the impact of political, socio-economic and cultural contexts on concerns about vaccination among HCPs is also necessary.

## Introduction

Vaccination is often considered as one of the most cost-effective public health intervention, leading to the elimination and control of infectious diseases and millions of lives being saved each year.[1], [2] Health care professionals (HCPs) are considered to be at significant risk for acquiring or transmitting of infectious diseases. Recommended vaccines for HCPs are influenza vaccine, Diphtheria, Tetanus and Pertussis (DTP) booster and Hepatitis B vaccine.[3] Vaccine hesitancy worldwide is a cause for concern and a major challenge for public health,[4], [5] contributing to drops in vaccine coverage along with an increasing risk of vaccine-preventable disease outbreaks and epidemics.[6] Studies are showing that although health HCPs are widely considered to be the most trusted source of information on vaccine-related topics, some report their own hesitancy around certain vaccines, influencing their intentions to vaccinate themselves as well as their recommendations to patients and target populations.[7], [8], [9], [10], [11], [12], [13], [14], [15], [16].

A 2022 literature review on vaccine hesitancy amongst HCPs in Europe determined that HCPs believe that vaccination is essential to protect both themselves and their patients.[17] Nevertheless, opinions that some diseases, for example influenza, are less risky were reported by a number of HCPs as a reason for not getting vaccinatated.[17] In most of the studies conducted, HCPs identified their concerns pertaining to both the short- and long-term side-effects. Additionally, certain studies drew attention to mistrust of health authorities and the pharmaceutical industry. [17].

While individual studies have looked at HCPs vaccine hesitancy in a local or national context, very few studies exist that aim to compare confidence levels across countries. The aim of this study was to investigate HCPs attitudes towards vaccination and their role in addressing vaccine hesitancy among patients in 12 European countries as well as Morocco, Turkey and Switzerland.

## Materials and methods

### Study design

A mixed-methods approach with a sequential explanatory design was used, in which qualitative data helped explain initial quantitative results.[18] An online cross-sectional survey was used to determine drivers of HCP vaccine confidence and examine how these drivers vary across nations. Subsequently, in-depth semi-structured interviews were conducted with a subsample of HCPs who completed the online survey to explore barriers to HCPs vaccine uptake and their role in addressing vaccine hesitancy among patients. Ethical approval was obtained from LSHTM observational research ethics committee (Ref:22805).

## Cross-sectional survey

### Setting and data collection

The cross-sectional survey was conducted among HCPs in 11 EU Member States: Austria, Belgium, France, Germany, Greece, Hungary, Italy, the Netherlands, Poland, Romania, and Spain as well as the United Kingdom (UK), Switzerland, Morocco, and Turkey. Approximately 100 HCPs in each country (a total of 1,504 HCPs) were involved in the survey. HCPs were recruited by ORB International Association (Gallup International) and their local partners. HCPs were invited via online panel providers to complete an online survey between 1 February 2021 and 29 March 2021. The surveys were conducted in local languages and findings were translated to English for the analysis.

### Survey development

The survey questions were developed by the Vaccine Confidence Project™ (VCP) and included questions from existing surveys[19], [20], [21] and the Vaccine Confidence Index™ (VCI) which has been used in previous global surveys on vaccine confidence.[22], [23], [24].

The survey comprised of five major sections.

1. **Socio-demographic characteristics of HCPs**: Gender (male or female), age (18–34, 35–44, 45–55, 56–64, 65+ years), profession (general practitioner (GP), pediatrician, nurse / midwife), country of residence, type of location (capital, big city, small city, village), and time in profession (up to 10 years, more than 10 years).
2. **Previous vaccination history of HCPs** (seasonal influenza for 2019/2020 and 2020/2021, Diphtheria, Tetanus and Pertussis (DTP) booster and Hepatitis B), and HCPs willingness to recommend vaccines against: Measles, Mumps, Rubella (MMR), Human Papillomavirus (HPV) and coronavirus (COVID-19) to patients. These were measured on 5-point Likert scales (1) highly likely, (2) somewhat likely, (3) somewhat unlikely, (4) highly unlikely and (5) don’t know.
3. **HCPs confidence when discussing vaccine safety**, the use of adjuvants, and the importance of vaccines with their patients. Participants responded to each statement using a 5-point Likert scale (1) very comfortable, (2) somewhat comfortable, (3) somewhat uncomfortable, (4) not at all comfortable, (5) don’t know. HCPs perceived role in encouraging patients to have a vaccination were measured on 5-point Likert scale (1) strongly agree, (2) tend to agree, (3) tend to disagree, (4) strongly disagree and (5) don’t know.
4. **HCPs perceptions of the importance, safety, effectiveness** and the compatibility of vaccines with their religious beliefs in general (not focusing on specific vaccines) as measured by the Vaccine Confidence Index (VCI). The VCI also measured perceptions of the importance, safety and effectiveness of four specific vaccines against: Measles, Mumps, Rubella (MMR), Human Papillomavirus (HPV), seasonal influenza, and coronavirus (COVID-19). Responses were measured using a 5-point Likert scale (1) strongly agree, (2) tend to agree, (3) tend to disagree, (4) strongly disagree and (5) don’t know.
5. Factors influencing vaccine confidence. Participants responded to each statement using a 5-point Likert scale (1) strongly agree, (2) tend to agree, (3) tend to disagree, (4) strongly disagree and (5) don’t know.

### Statistical analysis

Country-level frequencies and unweighted proportions are presented for the different categories of study variables. HCP’ perceptions towards the importance, safety, effectiveness and compatibility of vaccines with religious beliefs were dichotomized as “agree” (strongly agree/tend to agree) or “disagree” (strongly disagree/tend to disagree/ don’t know).

The study outcomes (dependent variables) analyzed were: 1) “Overall I think vaccines in general are safe” (agree or disagree), 2)” Overall I think vaccines in general are important for children to have” (agree or disagree) and 3)” Overall I think vaccines in general are effective” (agree or disagree). All other participant characteristics were considered covariates (independent variables) in the analyses.

Multivariable logistic regression was run on three dependent variables: A) model 1: NOT strongly agreeing with “vaccines in general are safe” vs DO, B) model 2: NOT strongly agreeing with “vaccines in general are important for children to have” vs DO and C) model 3: NOT strongly agreeing with “overall I think vaccines in general are effective” vs DO.

Logistic regression was used due to the high proportions of “strongly agree” responses. The regressions were run on predication of “lack of confidence” (e.g. NOT Strongly agreeing). The regressions were run in several stages.

1. Demographic screening according to country, gender, age, profession, whether urban/rural, time in profession, and number of children aged 0–17 and aged 18+, using a modified backward and forward stepwise algorithm.
2. Retained demographics from stage (1) were entered and similar screening then took place for the explanatory variables.

The BIC statistic was used to determine the optimal variables to retain at each stage to avoid the over-fitting that can result from a regular stepwise algorithm and to guard against inflation of reporting false positives. [25].

Multivariable logistic regressions were used to determine the social-demographic drivers of HCP vaccine confidence (such as gender, age, profession, etc) and examine how these drivers vary across nations. The results were measured using odds ratios (OR’s) and 95 % confidence intervals (CI’s). The statistical analyses were run using IBM SPSS Version 28.0.

## Qualitative study

### Participants and data collection

In the last section of the survey, study participants were asked if they were willing to be contacted for a follow-up in-depth interview. Thirty-minute in-depth interviews were conducted with 10 HCPs in a subset of three countries (France, Greece and Hungry), selected as they were identified through the survey as the three countries with the lowest levels of vaccine confidence among HCPs.

The in-depth, semi-structured interviews were conducted by telephone and in the language of the participant by ORB International, between 5 and 30 May 2021. With permission from the participant, these were digitally audio-recorded. To ensure confidentiality, all identifiers such as names or specific locations were removed and replaced by identification numbers and first-letter countries abbreviation allocated to each participant. Data were collected on awareness, views, attitudes and decision-making factors influencing vaccination confidence and recommendations. The topic guides for these interviews were developed based on a literature review [17]and preliminary analysis of the survey data to cover predefined topics and provide the necessary flexibility for the interview to be shaped by participant’s awareness, experiences and interests.

### Content analysis

Audio recordings from the interviews were translated into English and transcribed into the NVivo® software. A consistent format was used to transcribe the interviews, with each new speaker starting on a new line, and using commonly agreed transcript conventions. All files were secured, and password protected. Transcripts were transcribed verbatim and analysed thematically using the stages of data familiarization, coding, and theme identification and refinement.[26] NVivo® software 12 was used to assist in data management and analysis.

## Results

### Quantitative results

A total of 1,504 HCPs were surveyed across 15 countries (Austria, Belgium, France, Germany, Greece, Hungary, Italy, the Netherlands, Poland, Romania, Spain, the UK, Switzerland, Morocco, and Turkey). The proportion of male HCPs in this survey was 51 %. The survey also showed that 10 % of study participants were aged between 18 and 24 years old, 20 % between 35 and 44, 32 % between 45 and 55, 29 % between 56 and 64, and 10 % were 65 +. More than two-thirds (63 %) of HCPs were GPs. Overall, 85 % of participants reported spending more than ten years in their professions. More than a third (40 %) of HCPs were living in big cities (See Appendix 1, A).

### Previous vaccination history and willingness to recommend vaccines to patients

The proportion of HCPs who reported they had been vaccinated against seasonal flu in the *2019/2020* influenza season was 75 %, compared to 74 % for *the 2020*/2021 influenza season. The lowest rates of influenza vaccination uptake were among participants from Morocco (35 %), Turkey (53 %) and Greece (69 %). Slightly less than half (46 %) of study participants reported that their last DTP booster was less than 10 years ago. More than two third (79 %) of participants reported that they had received 3 or more doses of Hepatitis B vaccine. (See Appendix 1, B).

**Fig. 1:**
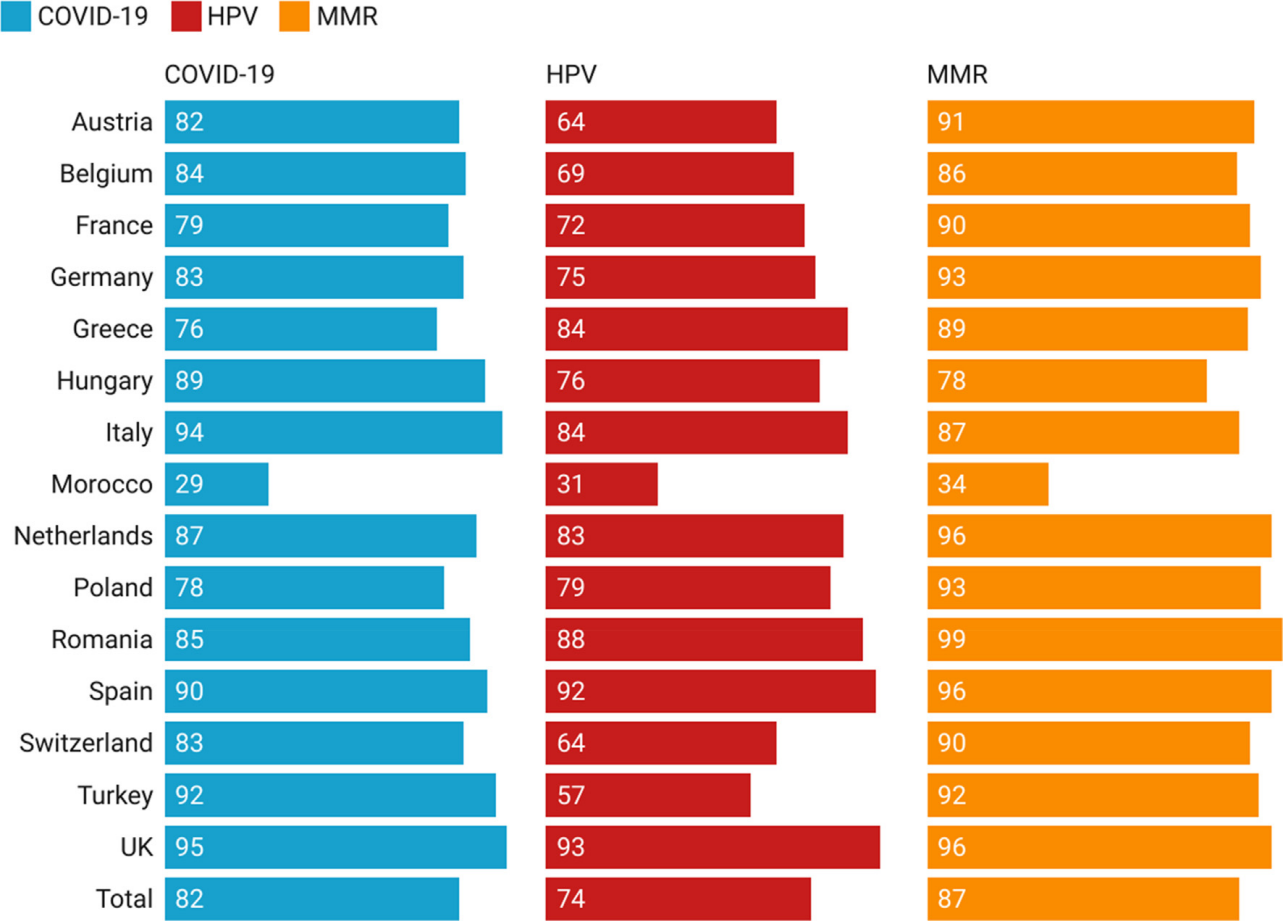
Percentage of HCPs who were highly likely willing to recommend MMR, HPV and COVID-19 vaccines to their patients.

The proportion of HCPs surveyed who reported being highly likely to recommend MMR, HPV and COVID-19 vaccines were 87 %, 74 % and 82 %, respectively. Among all 15 countries, Morocco had the lowest proportion of HCPs surveyed who stated being highly likely to recommend MMR (34 %), HPV (34 %) and COVID-19 (29 %) vaccines to their patients. (See Fig. 1).

The majority (87 %) of HCPs surveyed stated that they would definitely accept coronavirus (COVID-19) vaccine for themselves.

### Perceptions towards the importance, safety, effectiveness, and religious compatibility of vaccines.

**Map 1:**
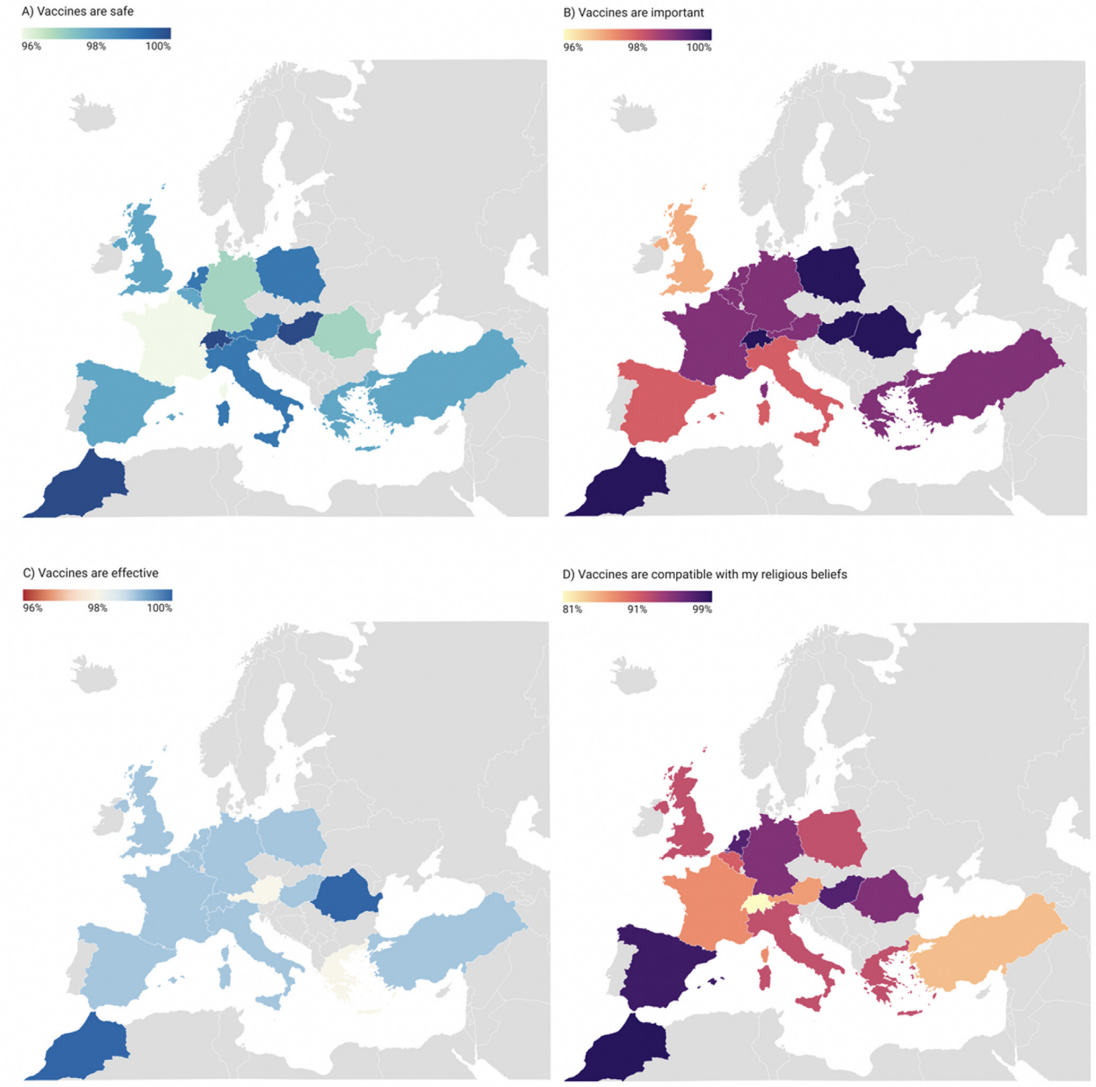
Percentage of HCPs in each country who agreed (strongly agreed or tended to agree) that vaccines in general are important, safe, effective and compatible with their religious beliefs.

**Fig. 2:**
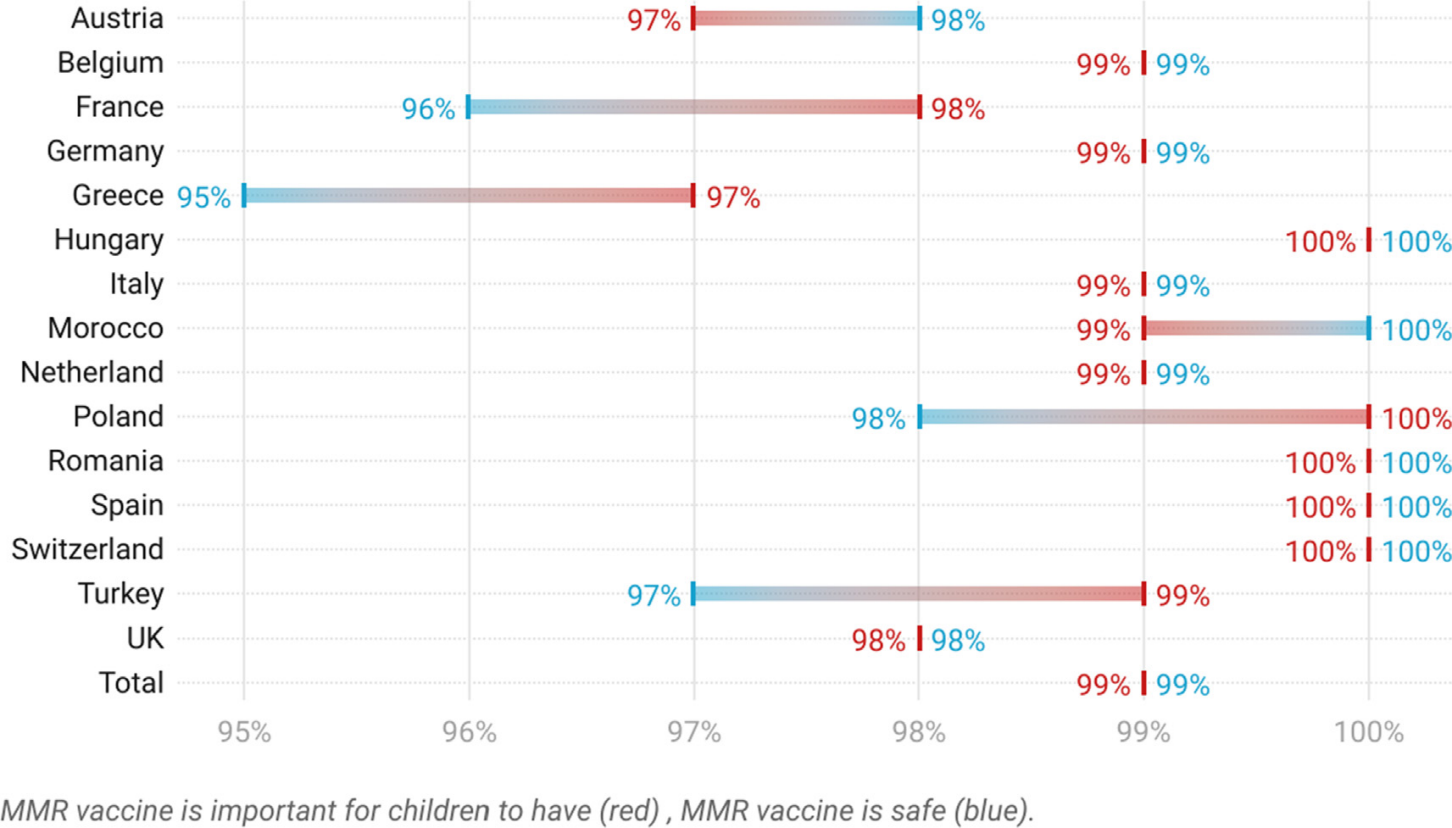
Percentage of HCPs in each country who agreed (strongly agreed or tended to agree) that the MMR vaccine is important and safe.

**Fig. 3:**
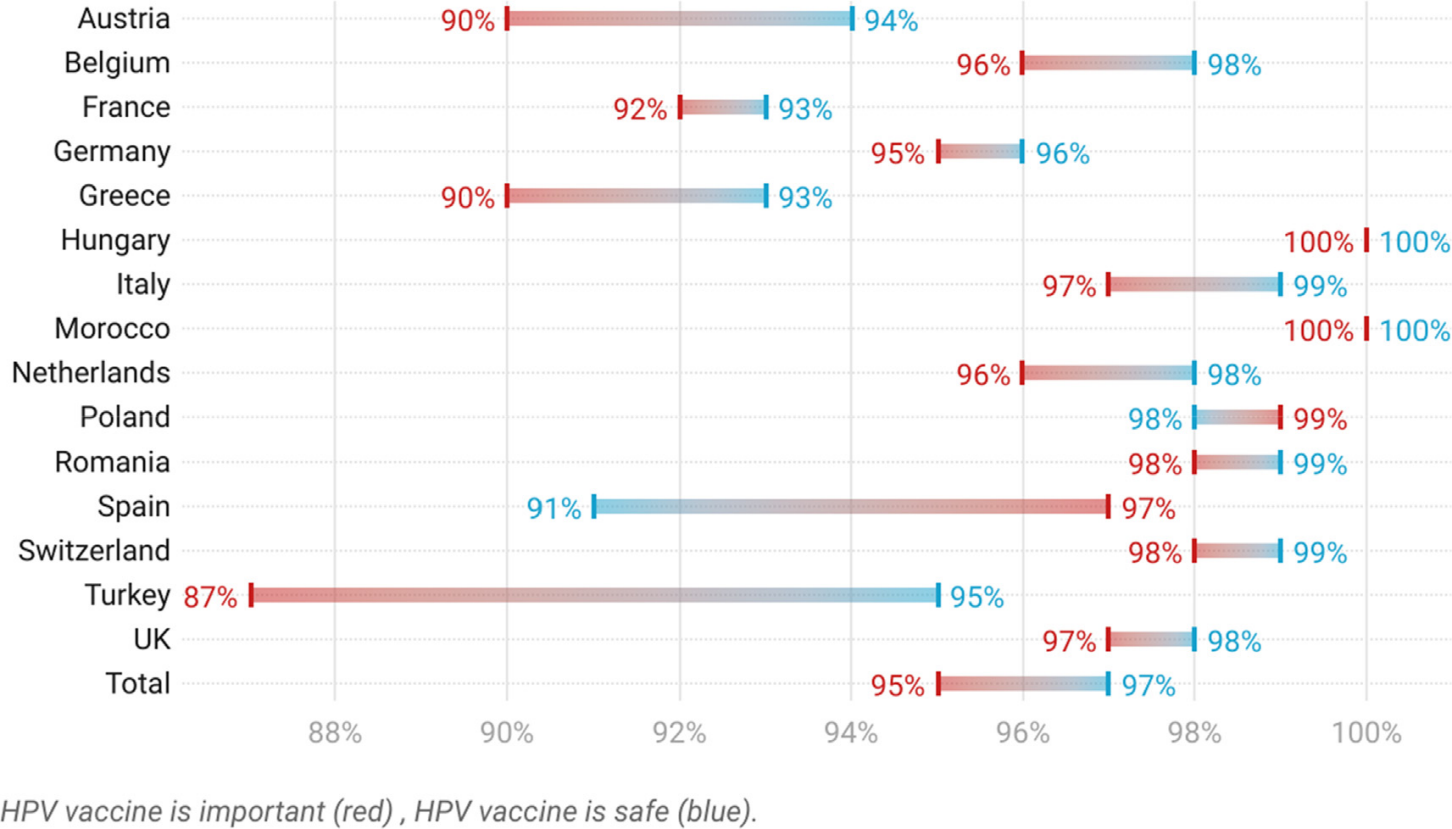
Percentage of HCPs in each country who agreed (strongly agreed or tended to agree) that the HPV vaccine is important and safe.

**Fig. 4:**
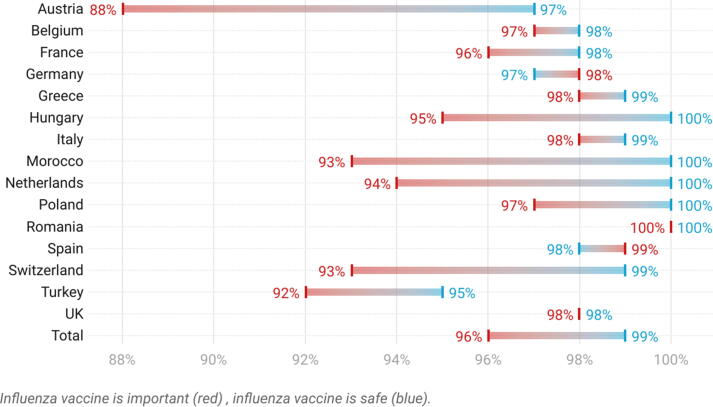
Percentage of HCPs in each country who agreed (strongly agreed or tended to agree) that the seasonal influenza vaccine is important and safe.

Across the 15 countries, HCP perceptions towards vaccination in general (not focusing on specific vaccines) were positive. A high proportion of the HCPs surveyed agreed (strongly agreed or tended to agree) that vaccines in general are important (99 %), safe (98 %), effective (99 %), and compatible with their religious beliefs (92 %) (See Map 1). Perceptions towards the importance, safety and effectiveness of four specific vaccines against: Measles, Mumps, Rubella (MMR), Human Papillomavirus (HPV), seasonal influenza, and coronavirus (COVID-19) are shown in Fig. 2, Fig. 3, Fig. 4 and appendix 1, C,D and E.).

## Drivers of lack of confidence in the safety, importance and effectiveness of vaccines in general

### Drivers of lack of confidence in vaccine safety

Drivers most consistently associated with lack of confidence in vaccine safety (“vaccines in general are safe”) were: disagreeing (OR 0.11, CI 0.05 – 0.24) or not being sure the COVID-19 vaccines are safe (OR 0.47, CI 0.23 – 0.92); not feeling “very comfortable” explaining the safety of vaccines to patients (OR 0.17, CI 0.09 – 0.33); tending to agree that children are vaccinated against too many diseases (OR 6.5, CI 3.6–11.9); tending to agree that new COVID-19 vaccines are important (OR 3.7, CI 1.4 – 6.8) and disagreeing / not being sure it is their role to encourage hesitant patients to get vaccinated (OR 0.55, CI 0.24 – 1.3) (Appendix 1, G). Additionally, being from Hungary (OR 9.4, CI 2.9–30.6), Italy (OR 7.1, CI 2.4–21.1), Romania (OR 5.9, CI 1.9–18), and Switzerland (OR 5, CI 1.6–15) was associated with lack of confidence in vaccine safety.

## Drivers of lack of confidence in vaccine importance for children

Drivers most consistently associated with lack of confidence in vaccine importance (“vaccines in general are important for children to have”) were: tending to agree children are vaccinated against too many diseases (OR 14.3, CI 6 – 34.3); not strongly agreeing that new COVID-19 vaccines are important (OR 0.33, CI 0.14 – 0.7); not feeling comfortable explaining the safety of vaccines to patients (OR 0.14, CI 0.06 – 0.34); being only 'somewhat' likely to recommend MMR vaccination (OR 1.8, CI 0.55 – 5.9) and being unlikely to recommend HPV vaccination (OR 0.2, CI 0.09 – 0.4). Moreover, being from Hungary (OR 13.2, CI 2.9 – 59.2) and being a nurse/midwife were also associated with lack of confidence in vaccine importance (Appendix 1, H).

### Drivers of lack of confidence in vaccine effectiveness

Drivers most consistently associated with lack of confidence vaccine effectiveness (“vaccines in general are effective”) were: not strongly agreeing COVID-19 vaccines are effective (OR 0.3, CI 0.14 – 0.7); not holding strong opinions about whether children vaccinated against too many diseases (OR 3.1, CI 2 – 4.9); not being 'very comfortable' explaining the value of vaccines to patients (OR 0.16, CI 0.06 – 0.4); not being 'highly likely' to recommend COVID-19 vaccines to patients (OR 0.4, CI 0.9 – 0.86); not 'strongly agreeing' that patients underestimate the individual benefits of vaccination (OR 0.5, CI 0.26–0.98); and being only somewhat likely to recommend MMR vaccination (OR 1.8, CI 0.76–4.4). Being from Hungary (OR 3.9, CI 1.2 – 11.9); and being a nurse/midwife were also associated with lack of confidence in vaccine effectiveness (Appendix 1, I).

### Encouraging patients to vaccinate and explaining the safety and value of vaccines

**Fig. 5:**
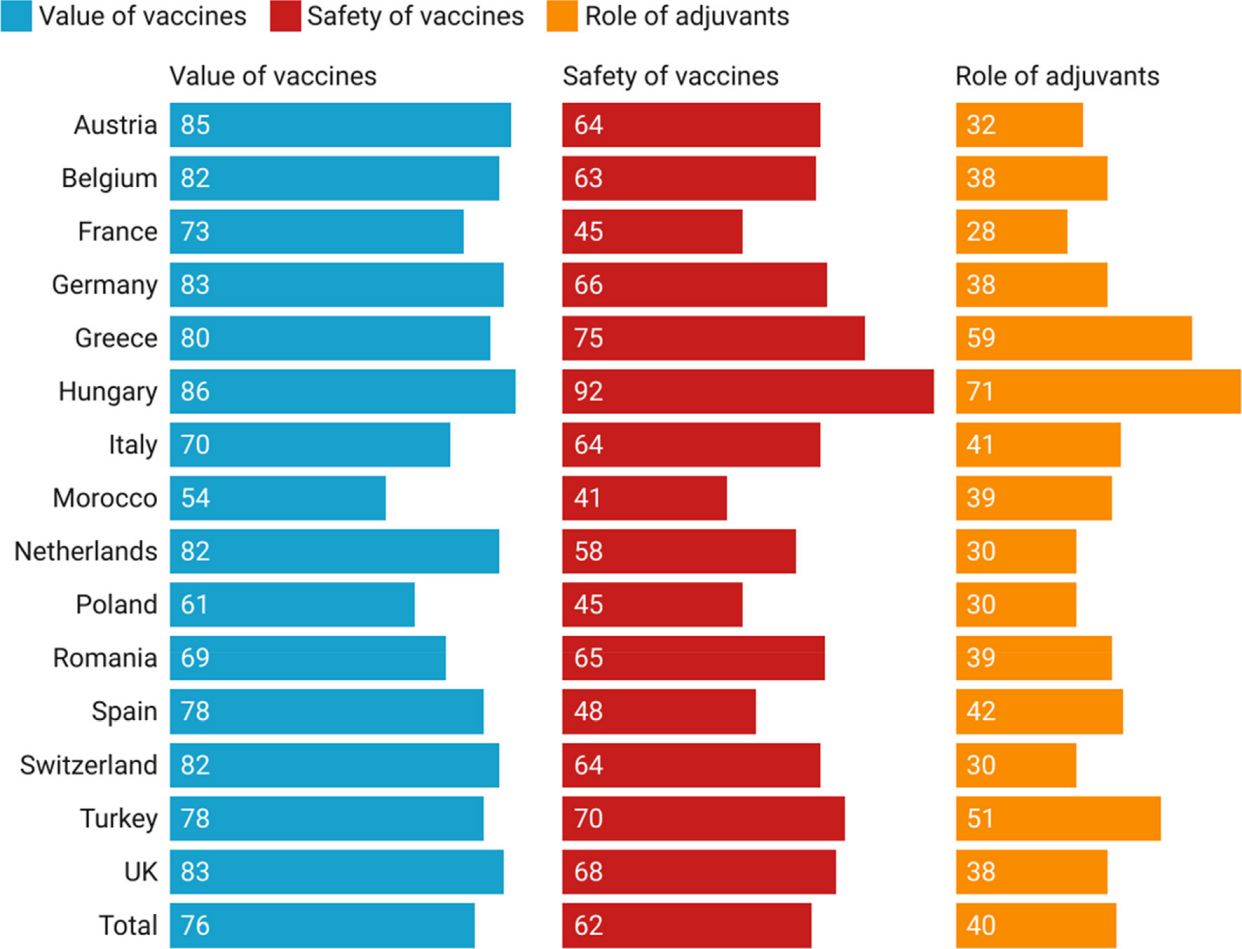
Percentage of HCPs in each country who felt very comfortable giving explanations to their patients about vaccine safety, value and the role of adjuvants.

More than two third (97 %) of HCPs surveyed agreed (agree or tend to agree) that it is their role to encourage their patients to have a vaccination even if they HCPs are hesitant. The proportions of HCPs surveyed who felt very comfortable giving explanations to their patients about vaccine safety, the value of vaccines, and the role of adjuvants were 62 %, 76 %, and 40 %, respectively (see Fig. 5).

### Trust in authorities’ recommendations, media messages, and the pharmaceutical industry

Only (12 %) of HCPs surveyed thought vaccines recommended by the authorities are pointless. A high percentage of HCPs (70 %) surveyed thought the media publishes too many negative messages about vaccination, but this was reported by only 9 % of surveyed HCPs in Morocco (See appendix 1, F). While only 15 % of HCPs surveyed stated that children are vaccinated against too many diseases, this belief was reported by 80 % of HCPs in Greece. Slightly more than one third (35 %) of HCPs surveyed believed that health authorities are influenced by the pharmaceutical industry, this was reported by 53 % HCPs in Turkey, 59 % in Greece, and 58 % in Belgium. More than one third (42 %) of HCPs surveyed stated that they trust their own judgement more than official recommendations. This was reported by a high percentage of HCPs in Turkey (77 %) and Romanian (77 %) (see appendix 1, F).

### Qualitative results

Thirty semi-structured interviews were conducted with HCPs,10 each from France, Greece and Hungary. Overall, 53 % of participants were male and they were aged between 44 and 70 years old. The majority were GPs (87 %), with dermatologists (7 %), oncologists and lung specialists (3 %) and internal medicine specialists (3 %) also participating.

### Barriers to vaccination uptake

An important barrier to seasonal influenza vaccination identified among study participants not routinely vaccinated against influenza was the perception that they are immune and that there is no need to get vaccinated every year “*I don’t see the need for me to get vaccinated every year*.” (F1). However, perceptions for other vaccines were sometimes different, as one HCP from Greece explained: “*Regarding the flu vaccine, I believe that I am somewhat immune. Regarding the other vaccines, I will follow the guidelines for myself in the same way I will do for my patients”* (G3). This immunity to influenza was explained to come from HCPs developing natural immunity by being exposed to viruses in work settings: “*we get so many viruses every day, and we have developed an adequate immune system*“(G5).

Some HCPs rejected vaccines because they believed they may cause side-effects. One HCP reported concerns about the safety of COVID-19 vaccine and the possibility of causing thrombosis “ *I admit that although I’m pro-vaccine and always been, I have major doubt here and I admit that I’m a bit scared*” (F7). Certain unvaccinated HCPS mentioned that they are exempted from vaccination (H5, F8): “*I have an autoimmune problem which is quite serious. It’s not that I don’t want to get vaccinated, it’s that I can’t*.” (F8). One HCP from Hungary prefers vaccines with a more modern technology that has no pathogen in it, and avoids traditional vaccines as those caused some body reactions (H5). Concern about side effects in relation to COVID-19 vaccination, particularly unknown long term side effects were reported by some HCPs in France and Greece but only one HCP from France reported this as a barrier to vaccination: “*Although it causes no issues in the short to medium term, or not many in any case, no one can say about the long term. It’s clear that no one can say”* (F10).

One HCP from France also raised the issue of cost and availability of vaccines: *“The barriers are the cost, when vaccines aren’t covered [by social insurance], [and] the availability when we’re told we’ll get the vaccines but desperately wait for them to arrive.*”. (F3).

Some HCPs reported that making vaccination mandatory for HCPs may have a negative impact on adopting vaccines (F3, G3). One French GP commented, “*I have a colleague who is totally opposed to vaccines. Making vaccination mandatory for HCPs will make him rebel even more, for example. He will be scandalised. So it won’t convince him more about vaccination*.” (F3). One interviewed HCP who declined receiving COVID-19 vaccine, believed that the vaccine has been used politically and that it has limited medical benefits. “*It’s political. It’s not medical. It has very, very little medical benefit”.* (F10).

The perception that HCPs were not at risk of COVID-19 because they did not have a chronic illness was also provided as a reason for refusing COVID-19 vaccination by HCPs: “*I’m not obese, I’m not diabetic, I don’t have hypertension, I’m in good health. I have been swimming in COVID for the last year. I’ve cared for lots of patients, I’ve taken samples, we didn’t have any masks at the start. I’ve always had negative serology. My body defends itself. It doesn’t get it. So, I don’t see the benefit for me in getting vaccinated. The benefit/risk balance isn’t pointing in the right direction” (*F10).

### Trust

HCPs in France, Greece and Hungry reported trusting vaccine information provided by health authorities, pharmaceutical companies, scientific articles or conferences. However, some criticism of information provided by Ministry of Health websites was raised, in particular about not being updated with the most up-to-date or required information: *“They are generally good, but they might be released a little late compared to the information we need. It’s a little too postponed” (F 2).*

A mistrust of health authorities was also observed among some of the interviewed HCPs in Hungary: ‘‘*Information is politicized in connection with the current vaccination program*.” (H5).

Some HCPs believed that pharmaceutical companies do not provide sufficient information about side-effects and that occasionally they over promote their products. Various HCPs believed that pharmaceutical companies and sales representatives are biased and have financial interests, or that companies are cautious not to damage their reputation by providing data that is different to what they initially provided in their studies. Sources such as mainstream media, newspapers, news channels were among those less trusted by HCPs.

### Quality and quantity of information

HCPs in France, Greece and Hungry were satisfied with the quantity and quality of information they received about vaccination through leaflets or websites. However, while some HCPs reported that they receive too much information (“*We are a bit submerged by information. So, too much information kills information*” (F1)), others reported a lack of information about vaccination “*In terms of quantity, there should be more. I feel there is not enough information*” (F5).

### Role of HCPs in responding to patient hesitancy

Nearly all interviewed HCPs believed that it is their role to give patients advice about vaccination and felt comfortable responding to vaccine hesitant parents: “*That’s one of the basic pillars of my job*” (F4). Some HCPs felt less comfortable recommending specific vaccines either because they felt they are less important “ *what I recommend a bit less is the vaccine against tick-borne encephalitis. I don’t think it would be absolutely necessary*” (H1) “ *for example, the rotavirus not so much*” (H6), or because they might cause some side effects” *I am not too enthusiastic when I have to administer the vaccines against encephalitis. As it has bigger possible risks*” (H6). Lack of knowledge about certain vaccines such as the vaccine for meningitis A,C,W was a reason for one HCP to feel less comfortable recommending them “I *feel less comfortable recommending them because I’m less familiar with them*” (F4). The same HCP also felt less comfortable recommending non– mandatory vaccines which are more exotic or those which are not reimbursed *“ Yes: in addition, the vaccine for herpes zoster is not reimbursed”.*

One HCP from France stressed that HCPs should be convinced about vaccines themselves and be well informed regarding study results and vaccine safety “*what we need is to be convinced ourselves, first of all*” (F7). Another HCP from Greece commented that it is not their role to respond to parents who are reluctant to vaccinate themselves or their children and that this is the responsibility of the government: “*I think it's a waste of time to sit down and talk to them, because they're going to reproduce things that don't have a logical continuity and consequence, so I think it's a waste of time to deal with them. That's a matter for the state to solve*” (G6).

Various HCPs explained they have to attempt to influence patients’ decision-making regarding vaccination by sharing professional experiences “ *I use my experience. I tell them, I’ve been giving hepatitis B vaccines for 30 years. I ‘ve never had any problems*” (F7), or explaining that vaccination is mandatory for children to go to school(F5, F8,F10). One HCP from France admitted that certain HCPs may find it more straightforward to provide fake vaccination certificates than to make an effort to persuade hesitant parents “*I think some physicians make fake certificates. But that’s their problem*” (F8).

## Discussion

Our mixed methods study was conducted to investigate HCP attitudes towards vaccination. A lack of confidence in vaccine safety, importance and effectiveness was found among HCPs from Hungary. HCPs from Italy, Romania and Switzerland were also found to be less confident in vaccines safety. Concerns about side effects were mentioned as a reason for not supporting universal varicella vaccination by 28 % of HCPs in Hungary. [27] A study in Italy found that 59.3 % of HCPs missed Hepatitis B vaccination because they believed the vaccine was needless, compared to 10.4 % for MMR vaccination and 20.3 % for DTP vaccination.[28] Concerns about vaccine safety were also reported in previous qualitative studies among HCPs in Romania and Greece.[14] A European literature review found that one of the most commonly mentioned barriers to receiving or recommending vaccination in general among HCPs in Europe pertains to the perceived lack of effectiveness, doubts regarding usefulness and the importance of particular vaccines, together with concerns with respect to vaccine safety.[17].

Influenza vaccination uptake was the lowest among our participants from Morocco (35 %), Turkey (53 %) and Greece (69 %). Through in-depth interviews with HCPs from France, Greece and Hungary, we found that barriers to influenza vaccination included not feeling at risk because of perceived natural immunity or not having a chronic disease, vaccine side effects, and the hassle of having to be vaccinated every year. The findings confirm those from a multicentre study conducted in Turkey, in which 53.1 % of unvaccinated HCPs reported that they do not think the influenza vaccine is necessary, 16 % feared the side-effects of the vaccine and 23 % believed they have a strong immune system.[29] In an additional study undertaken in Turkey, the perceived necessity to be vaccinated each year had a negative effect on HCPs vaccination behaviour.[30] A previous report also showed a low influenza vaccine coverage among Moroccan HCPs in 2016, with some HCPs raising concerns about varying influenza vaccine efficacy.[31] In order to address these misconceptions identified among HCPs, educational strategies are essential [31] Interventions and campaigns to improve confidence and vaccination uptake among HCPs in general and specifically for influenza vaccines should address several of the key concerns that have been identified in this study as well as in previous studies, such as the perceived lack of effectiveness of particular vaccines and concerns about the side-effects but also emphasise the part HCPs play in the transmission of infections to patients and the significance of HCPs being vaccinated to protect patients. [17].

The study also indicated that nurses and midwives were less confident in the importance and effectiveness of vaccines. Inadequate knowledge regarding vaccination among nurses and midwives may be one of the factors affecting vaccines confident among this group of HCPs. A study was conducted among health care providers in 23 countries reported that compared to physicians, community health workers and other healthcare providers reported higher degrees of vaccine hesitancy. [32] Improving confidence among nurses and midwives is important as previous studies showed that perceived lack of effectiveness was one of the most common citied barrier to accepting or recommending vaccines among nurses and midwives.[11], [33], [34], [35]. As levels of vaccine confidence and the factors impacting vaccine acceptance can differ between types of HCPs, strategies to improve vaccination coverage should be targeted to different HCP groups.[11], [17].

In our study, Morocco had the lowest rates of HCPs who were highly likely to recommend MMR, HPV or Covid-19 vaccines. Lack of trust in Covid-19 vaccines and health authorities was reported by 22 % of Moroccans in 2021.[36] HCPs should play a crucial role in providing education and awareness regarding vaccination to their patients. Political, socio-economic and cultural factors may play a role in decreasing HCPs willing to recommend some vaccines to their patients. Studies investigating the impact of political, socio-economic and cultural circumstances on concerns about vaccination among HCPs in Morocco and their willingness to recommend vaccination are vital.[14].

More than one third of HCPs in this study reported a lack of trust in health authorities and in the information they provide. A previous study in Greece showed similar high-level of mistrust in health authorities among HCPs, which was in part explained by the political and economic context in which the study took place.[14] A study from France revealed that GPs who conveyed a stronger trust in institutions were found to be significantly more likely to recommend vaccines, showing the importance of building HCPs trust in health authorities.[37].

As our interviews have shown, some HCPs may issue fake vaccination cards for themselves or their patients, posing the risk of the spread of infectious diseases and providing false public health data to health policy makers. This also questions the ethics of some HCPs, and the reliance on them to improve patient vaccine hesitancy. More attempts to improve confidence in vaccination are required for both the general population and HCPs.

Distrust of pharmaceutical companies was also shown in this study, with concerns raised with respect to their financial interests, along with concerns as regards a lack of transparency, for instance in relation to reporting some side-effects. Notwithstanding that the pharmaceutical industry works in highly regulated environments and adheres to high quality standards with regards to vaccine development and manufacture, improving communication and transparency processes may possibly assist with tackling concerns among both professionals and the public.[38].

This study consisted of a mixed methods approach that has allowed us to better understand the barriers to HCPs vaccine uptake and their role in addressing patient vaccine hesitancy. Findings from the study should be interpreted in light of a number of limitations. While we selected countries for qualitative interviews based on confidence levels, we did not conduct in-depth qualitative interviews in countries low vaccination coverage or less willingness to recommend vaccination to patients, e.g., Morocco and Turkey. Further data collection in these countries is required to explore the drivers of and barriers to HCPs vaccine uptake and recommendations. This may extend beyond Morocco and Turkey and include other countries in the Middle East and North Africa. A further limitation regarding our methodology is that the qualitative data collection in France, Greece and Hungry was based on a list of participants from these countries who agreed to be contacted for follow up in-depth interviews and was not based on a prior selection criteria from the survey e.g. those who were unvaccinated or those who were less willing to recommend vaccines to patients. This may have limited our understanding of barriers to vaccination and recommendation as the majority of the HCPs selected for the in-depth interviews were vaccinated and willing to recommend vaccines to their patients. Therefore the views of the HCPs interviewed in this study must be interpreted with caution. Similarly, in our survey analysis, we used logistic regression analysis instead of ordinal logistic regression, which in turn, may have prevented us from capturing some nuances between strongly agree and tend to agree by combining both in our logistic regression. The logistic regression analysis was used due to high proportions of strongly agree responses.

This study has corroborated the existence of vaccine hesitancy among HCPs in some countries such as Italy, Hungary, Romania, Switzerland, Morocco and Turkey. HCPs raised concerns pertaining to the side effects of vaccination and conveyed a lack of trust in health authorities. It is essential that vaccine confidence among HCPs is improved, given that they have been revealed to have the capacity to impact vaccination uptake by patients. Educational strategies to improve confidence in vaccines focus on these concerns and adjusted to the specific political, social, cultural and economic context of the country are therefore essential. More comprehensive, context-specific interventions are needed to address trust in health systems and healthcare workers’ perceived roles and level of confidence in responding to patient hesitancy.

**Contributors**

HJL and EK conceived and designed the study. AKA conducted the statistical analyses and created the figures. AKA and EK conducted the thematic analysis. AKA drafted the first version of the manuscript. All authors contributed to data interpretation, and finalised and approved the manuscript.

Declaration of interests

Authors of this paper are part of research projects funded by GlaxoSmithKline Plc (GSK) (HJL/EK), AstraZeneca (HJL, EK, AA), Merck Sharp & Dohme ltd (Merck) (HJL/EK/AA) and Janssen (HJL/EK); and received support for participating in Merck and GSK meetings (HJL/EK). HJL is a member of the Merck Vaccine Confidence Advisory Board.

## Declaration of Competing Interest

The authors declare the following financial interests/personal relationships which may be considered as potential competing interests: [Abrar Alasmari reports financial support was provided by Merck Sharp & Dohme UK ltd. Heidi Larson reports a relationship with Merck Sharp & Dohme UK ltd that includes: board membership. Heidi Larson - Emilie Karafillakis reports a relationship with Merck Sharp & Dohme UK ltd and GSK that includes: consulting or advisory. Authors of this paper are part of other research projects funded by GlaxoSmithKline Plc (GSK) (HJL/EK), AstraZeneca (HJL, EK, AA), Merck Sharp & Dohme ltd (Merck) (HJL/EK/AA) and Janssen (HJL/EK); and received support for participating in Merck and GSK meetings (HJL/EK). HJL is a member of the Merck Vaccine Confidence Advisory Board.].

## Data Availability

Data will be made available on request.

## References

[1] Greenwood B. (2014). The contribution of vaccination to global health: past, present and future. Philosophical Transactions of the Royal Society B: Biological Sciences.

[2] Ehreth J. (2003). The global value of vaccination. Vaccine.

[3] CDC. Recommended Vaccines for Healthcare Workers [Internet]. CDC; 2016 [cited 2022 May]. Available from: https://www.cdc.gov/vaccines/adults/rec-vac/hcw.html.

[4] Larson H.J., Schulz W.S., Tucker J.D., Smith D.M. (2015). Measuring vaccine confidence: introducing a global vaccine confidence index. PLoS currents.

[5] Shetty P. (2010). Experts concerned about vaccination backlash. The Lancet.

[6] Dubé E., Laberge C., Guay M., Bramadat P., Roy R., Bettinger J.A. (2013). Vaccine hesitancy: an overview. Human vaccines & immunotherapeutics.

[7] Benninghoff B., Pereira P., Vetter V. (2020). Role of healthcare practitioners in rotavirus disease awareness and vaccination–insights from a survey among caregivers. Human Vaccines & Immunotherapeutics.

[8] Czajka H., Czajka S., Biłas P., Pałka P., Jędrusik S., Czapkiewicz A. (2020). Who or what influences the individuals’ decision-making process regarding vaccinations?. Int J Environ Res Public Health.

[9] Kilich E., Dada S., Francis M.R., Tazare J., Chico R.M., Paterson P. (2020). Factors that influence vaccination decision-making among pregnant women: a systematic review and meta-analysis. PLoS ONE.

[10] De Figueiredo A., Karafillakis E., Larson H. (2020).

[11] Durovic A., Widmer A.F., Dangel M., Ulrich A., Battegay M., Tschudin-Sutter S. (2020). Low rates of influenza vaccination uptake among healthcare workers: Distinguishing barriers between occupational groups. Am J Infect Control.

[12] Attwell K., Wiley K., Waddington C., Leask J., Snelling T. (2018). Midwives’ attitudes, beliefs and concerns about childhood vaccination: a review of the global literature. Vaccine.

[13] Paterson P., Meurice F., Stanberry L.R., Glismann S., Rosenthal S.L., Larson H.J. (2016). Vaccine hesitancy and healthcare providers. Vaccine.

[14] Karafillakis E., Dinca I., Apfel F., Cecconi S., Wűrz A., Takacs J. (2016). Vaccine hesitancy among healthcare workers in Europe: A qualitative study. Vaccine.

[15] Dini G., Toletone A., Sticchi L., Orsi A., Bragazzi N.L., Durando P. (2018). Influenza vaccination in healthcare workers: A comprehensive critical appraisal of the literature. Human vaccines & immunotherapeutics.

[16] Lin C., Mullen J., Smith D., Kotarba M., Kaplan S.J., Tu P. (2021). Healthcare providers’ vaccine perceptions, hesitancy, and recommendation to patients: a systematic review. Vaccines.

[17] Pavlovic D., Sahoo P., Larson H., Karafillakis E. (2022). Factors influencing healthcare professionals’ confidence in vaccination in Europe: a literature review. Human Vaccines & Immunotherapeutics.

[18] Qualitative C.JW. (2018).

[19] Verger P., Dualé C., Lenzi N., Scronias D., Pulcini C., Launay O. (2021). Vaccine hesitancy among hospital staff physicians: A cross-sectional survey in France in 2019. Vaccine.

[20] Verger P., Collange F., Fressard L., Bocquier A., Gautier A., Pulcini C. (2016). Prevalence and correlates of vaccine hesitancy among general practitioners: a cross-sectional telephone survey in France, April to July 2014. Eurosurveillance.

[21] Verger P., Fressard L., Collange F., Gautier A., Jestin C., Launay O. (2015). Vaccine hesitancy among general practitioners and its determinants during controversies: a national cross-sectional survey in France. EBioMedicine.

[22] Larson H, de Figueiredo A, Karafillakis E, Rawal M. State of vaccine confidence in the EU 2018. Luxembourg: Publications Office of the European Union. 2018;10:241099.

[23] Larson H.J. (2018). The state of vaccine confidence. The Lancet.

[24] Larson H.J., Jarrett C., Schulz W.S., Chaudhuri M., Zhou Y., Dube E. (2015). Measuring vaccine hesitancy: The development of a survey tool. Vaccine.

[25] Gelman A., Hwang J., Vehtari A. (2014). Understanding predictive information criteria for Bayesian models. Statistics and computing.

[26] Braun V., Clarke V. (2006). Using thematic analysis in psychology. Qualitative research in psychology.

[27] Huber A., Gazder J., Dobay O., Mészner Z., Horváth A. (2020). Attitudes towards varicella vaccination in parents and paediatric healthcare providers in Hungary. Vaccine.

[28] Di Martino G., Di Giovanni P., Di Girolamo A., Scampoli P., Cedrone F., D’Addezio M. (2020). Knowledge and attitude towards vaccination among healthcare workers: a multicenter cross-sectional study in a Southern Italian Region. Vaccines.

[29] Korkmaz N., Nazik S., Gümüştakım R.Ş., Uzar H., Kul G., Tosun S. (2021). Influenza vaccination rates, knowledge, attitudes and behaviours of healthcare workers in Turkey: A multicentre study. Int J Clin Pract.

[30] Akan H., Yavuz E., Yayla M., Külbay H., Kaspar E., Zahmacıoğlu O. (2016). Factors affecting uptake of influenza vaccination among family physicians. Vaccine.

[31] Mantel C, Chu SY, Hyde TB, Lambach P, Group IPI. Seasonal influenza vaccination in middle-income countries: Assessment of immunization practices in Belarus, Morocco, and Thailand. Vaccine. 2020;38(2):212-9. 10.1016/j.vaccine.2019.10.028 PMC6961110 31699507

[32] Leigh J.P., Moss S.J., White T.M., Picchio C.A., Rabin K.H., Ratzan S.C. (2022). Factors affecting COVID-19 vaccine hesitancy among healthcare providers in 23 countries. Vaccine.

[33] Flanagan P., Dowling M., Gethin G. (2020). Barriers and facilitators to seasonal influenza vaccination uptake among nurses: A mixed methods study. J Adv Nurs.

[34] Loubet P., Nguyen C., Burnet E., Launay O. (2019). Influenza vaccination of pregnant women in Paris, France: Knowledge, attitudes and practices among midwives. PLoS ONE.

[35] Von Perbandt E.D., Hornung R., Thanner M. (2018). Influenza vaccination coverage of health care workers: A cross-sectional study based on data from a Swiss gynaecological hospital. GMS infectious diseases.

[36] Statista. Leading reasons people would refuse to be vaccinated against coronavirus (COVID-19) in Morocco as of 2021. [Internet]. statista; 2021 [cited 2022 May]. Available from: https://www.statista.com/statistics/1224623/reasons-people-would-not-get-the-covid-19-vaccine-in-morocco/.

[37] Raude J., Fressard L., Gautier A., Pulcini C., Peretti-Watel P., Verger P. (2016). Opening the ‘Vaccine Hesitancy’black box: how trust in institutions affects French GPs’ vaccination practices. Expert review of vaccines.

[38] Black S., Rappuoli R. (2010).

